# Adsorptive Separation of Chlorobenzene and Chlorocyclohexane by Nonporous Adaptive Crystals of Perethylated Pillar[6]arene

**DOI:** 10.3390/molecules30153312

**Published:** 2025-08-07

**Authors:** Sha Wu, Yuyue Chi, Qian Dong, Jiong Zhou

**Affiliations:** 1 Department of Chemistry, College of Sciences, Northeastern University, Shenyang 110819, China; 2200283@stu.neu.edu.cn (S.W.); 2300286@stu.neu.edu.cn (Y.C.); 2270038@stu.neu.edu.cn (Q.D.); 2 Key Laboratory of Functional Molecular Solids, Ministry of Education, School of Chemistry and Materials Science, Anhui Normal University, Wuhu 241002, China

**Keywords:** pillararene, nonporous adaptive crystals, adsorptive separation, molecular recognition, supramolecular chemistry

## Abstract

The separation of chlorobenzene (**CB**) and chlorocyclohexane (**CCH**) using traditional industrial separation technologies (distillation, fractionation, and rectification) is a great challenge due to their close boiling points. Here, we report an innovative method for the separation of the mixture of **CB** and **CCH** by nonporous adaptive crystals (NACs) of perethylated pillar[6]arene (**EtP6**). NACs of **EtP6** (**EtP6***α*) can selectively adsorb **CCH** vapor from the vapor mixture of **CB** and **CCH** (*v*:*v* = 1:1) with a purity of 99.5%. Furthermore, **EtP6***α* can be recycled for five times without a significant loss of performance.

## 1. Introduction

The separation of aromatic compounds from their cyclic aliphatic counterparts, such as benzene/cyclohexane, toluene/methylcyclohexane, and chlorobenzene/chlorocyclohexane, is a critical industrial challenge. These cyclic aliphatics are predominantly produced via the catalytic hydrogenation of their corresponding aromatics [1,2,3,4,5,6]. Focusing on **CB**, this chlorinated aromatic is widely employed as a solvent, reagent, and intermediate in the chemical industry. However, **CB** poses significant environmental and health risks due to its persistence, bioaccumulation potential, and acute toxicity [7,8,9]. Consequently, converting **CB** into less harmful or reusable products like **CCH** through hydrogenation or electrochemical degradation is essential prior to disposal [10,11,12]. A major obstacle in this conversion process is the separation of unreacted **CB** from the product **CCH**. Their similar boiling points (**CB**: 132 °C; **CCH**: 142 °C) render conventional separation techniques like distillation highly energy-intensive for obtaining high-purity **CCH** [13,14,15,16]. Thus, it is essential to explore innovative and energy-saving separation strategies for aromatic and cyclic aliphatic compounds.

In recent decades, the use of porous materials like zeolites, metal–organic frameworks, and covalent organic frameworks for adsorptive separation has attracted much attention as an alternative strategy, which provides new inspiration and guidance for the development and innovation of traditional industrial separation technologies [17,18,19,20,21,22,23]. Although porous materials are commonly utilized as adsorbents for adsorptive separation, the instability in the recycling process has limited their further development [24,25,26,27,28]. Therefore, it is necessary to explore efficient and stable adsorptive separation materials.

Nonporous adaptive crystals (NACs) represent a novel category of supramolecular solid adsorbents first proposed and defined by Huang and co-workers [29]. In contrast to traditional porous materials with the Brunauer–Emmett–Teller (BET) specific surface area greater than 100 m^2^/g, NACs are nonporous in the un-adsorbed state with a BET-specific surface area of less than 10 m^2^/g. However, the intrinsic pores of NACs can be induced by specific guest molecules, which gives NACs great potential in adsorptive separation [30,31,32,33,34,35,36,37,38]. Moreover, NACs have the advantages of a simple preparation process, good chemical and thermal stability, and high recyclability [39,40,41,42,43,44,45,46,47]. At present, NACs based on various macrocycles, including pillararenes, biphenarenes, hybridarenes, and geminiarenes, have been applied in the field of adsorptive separation [48,49,50,51,52,53,54,55]. However, to the best of our knowledge, pillararenes-based NACs for the separation of **CB** and **CCH** have not been studied.

**Figure 1:**
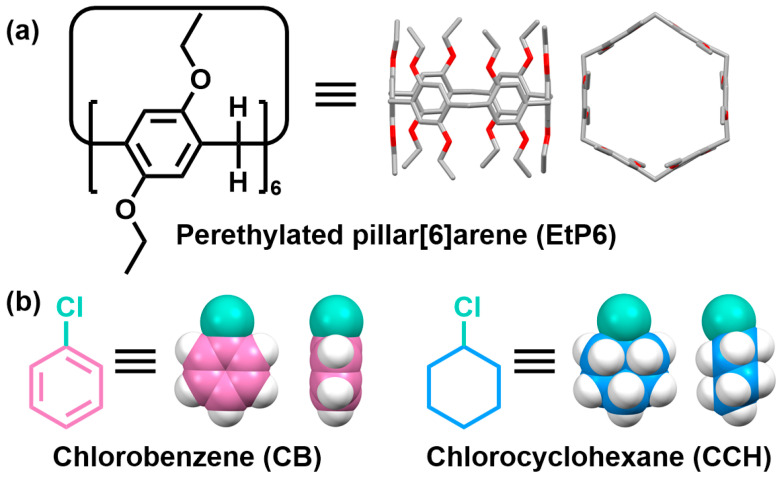
Chemical structures of (**a**) perethylated pillar[6]arene (**EtP6**) and (**b**) chlorobenzene (**CB**) and chlorocyclohexane (**CCH**).

Herein, we investigated the possibility of separating **CB** and **CCH** using perethylated pillar[6]arene (**EtP6**)-based NACs (Figure 1). NACs of **EtP6** (**EtP6**α) could selectively adsorb **CCH** vapor from the vapor mixture of **CB** and **CCH** (*v*:*v* = 1:1) with a purity of 99.5%. The experimental and computational results showed that the excellent selectivity of **EtP6**α to **CCH** vapor was due to the thermodynamic stability of the host–guest complex formed by **EtP6** and **CCH**. Additionally, the guest molecule loaded in **EtP6** could be removed by heating, transforming the crystal structure to its original guest-free state, thus enabling the reuse of **EtP6**α.

## 2. Results

The guest-free **EtP6** (**EtP6**α) was prepared according to the previously reported method [56,57,58,59,60]. ^1^H nuclear magnetic resonance (^1^H NMR) and thermogravimetric analysis (TGA) showed that the solvent was completely removed in **EtP6**α (Figures S1 and S2). The powder X-ray diffraction (PXRD) experiment showed that **EtP6**α was crystalline (Figure S3).

**Figure 2:**
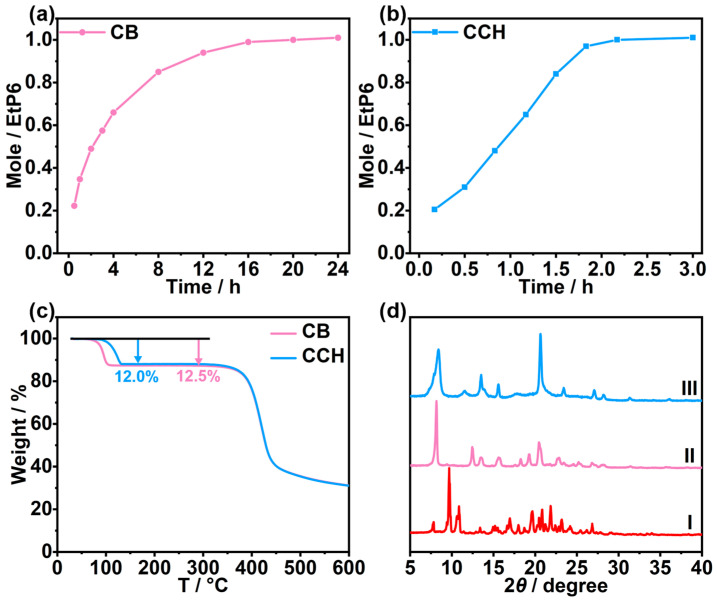
Time-dependent solid–vapor adsorption plots of **EtP6**α after adsorption of (**a**) **CB** vapor and (**b**) **CCH** vapor. (**c**) TGA of **EtP6**α after adsorption of **CB** vapor for 24 h and **CCH** vapor for 3 h. (**d**) PXRD patterns of (I) **EtP6**α and (II) **EtP6**α after adsorption of **CB** vapor; (III) **EtP6**α after adsorption of **CCH** vapor.

The adsorption properties of **EtP6**α toward **CB** vapor and **CCH** vapor were studied by ^1^H NMR, TGA, and PXRD. Both single-component **CB** vapor and **CCH** vapor could be adsorbed by **EtP6**α, and the proton signal peaks of **CB** or **CCH** appeared in ^1^H NMR of **EtP6**α after the adsorption of **CB** vapor or **CCH** vapor, while the proton signal peaks of **EtP6**α did not show significant change (Figures S6 and S7). Notably, the adsorption amounts of **EtP6**α for **CB** vapor and **CCH** vapor both increased with time, reaching saturation points at 24 h and 3 h, respectively (Figure 2a,b). Moreover, the adsorption amount was one **CB** molecule per **EtP6** molecule or one **CCH** molecule per **EtP6** molecule calculated by the ^1^H NMR spectra (Figures S4–S7). The adsorption amounts of **CB** and **CCH** by **EtP6**α were further confirmed by TGA. The weight loss of **EtP6**α after the adsorption of **CB** vapor and **CCH** vapor was 12.5% and 12.0%, respectively, indicating that one **EtP6** molecule could adsorb one **CB** molecule or one **CCH** molecule (Figure 2c, Figures S8 and S9). These results revealed that **EtP6**α had good capacity to adsorb **CB** vapor and **CCH** vapor. Furthermore, the PXRD patterns of **EtP6**α after the adsorption of **CB** vapor or **CCH** vapor changed significantly, while they were different from each other (Figure 2d, Figures S10 and S11). These results proved that **EtP6**α could adsorb **CB** vapor or **CCH** vapor with crystalline transitions from **EtP6**α to **CB**-loaded **EtP6** (**CB**@**EtP6**) or **CCH**-loaded **EtP6** (**CCH**@**EtP6**).

**Figure 3:**
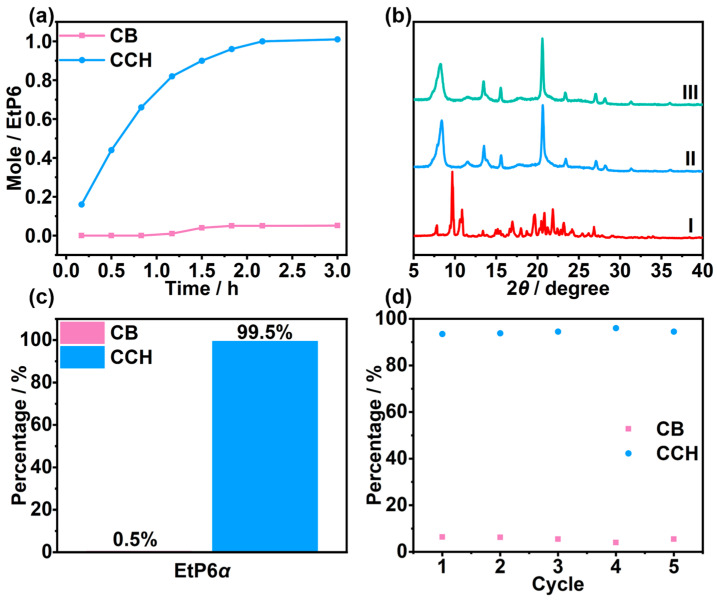
(**a**) Time-dependent solid–vapor adsorption plots of **EtP6**α after adsorption of the vapor mixture of **CB** and **CCH** (*v*:*v* = 1:1). (**b**) PXRD patterns of (I) **EtP6**α and (II) **EtP6**α after adsorption of **CCH** vapor; (III) **EtP6**α after adsorption of the vapor mixture of **CB** and **CCH** (*v*:*v* = 1:1). (**c**) Relative amounts of **CB** and **CCH** adsorbed by **EtP6**α after 3 h as measured by HS-GC. (**d**) Relative amounts of **CB** and **CCH** adsorbed by **EtP6**α after five cycles.

The selective adsorption capacity of **EtP6**α for the vapor mixture of **CB** and **CCH** (*v*:*v* = 1:1) was studied in light of its adsorption capacity for **CB** vapor and **CCH** vapor. According to the time-dependent adsorption plots of **EtP6**α for the vapor mixture of **CB** and **CCH** (*v*:*v* = 1:1), the adsorption amount of **CCH** by **EtP6**α increased with time, reaching the saturation point after 3 h (Figure 3a). In contrast, the adsorption amount of **CB** by **EtP6**α was negligible. The adsorption amount was one **CCH** molecule per **EtP6** molecule calculated by ^1^H NMR and TGA (Figures S12 and S13). Moreover, the PXRD pattern of **EtP6**α after the adsorption of the vapor mixture of **CB** and **CCH** (*v*:*v* = 1:1) changed and was consistent with that of **EtP6**α after the adsorption of **CCH** vapor alone (Figure 3b and Figure S14). These results indicated that **EtP6**α selectively adsorbed **CCH** vapor from the vapor mixture of **CB** and **CCH** (*v*:*v* = 1:1), and the crystal structure of **EtP6**α was transformed into **CCH**@**EtP6**.

According to the head space gas chromatography (HS-GC) experiment, 99.5% of **CCH** was loaded in **EtP6**α, whereas 0.5% of **CB** was loaded in **EtP6**α (Figure 3c and Figure S15). This result revealed that **EtP6**α could separate 99.5% of pure **CCH** vapor from the vapor mixture of **CB** and **CCH** (*v*:*v* = 1:1), with a higher separation purity than previously reported for geminiarene (97.5%) (Table S3). Furthermore, the recyclability of **EtP6**α was investigated by cyclic adsorption experiments. The **CCH**-loaded **EtP6**α could release **CCH** to reactivate upon heating at 120 °C under vacuum. The reactivated **EtP6**α could be reused to selectively adsorb **CCH** vapor from the vapor mixture of **CB** and **CCH** (*v*:*v* = 1:1). Notably, **EtP6**α showed no obvious loss of performance after recycling five times (Figure 3d and Figure S16).

**Figure 4:**
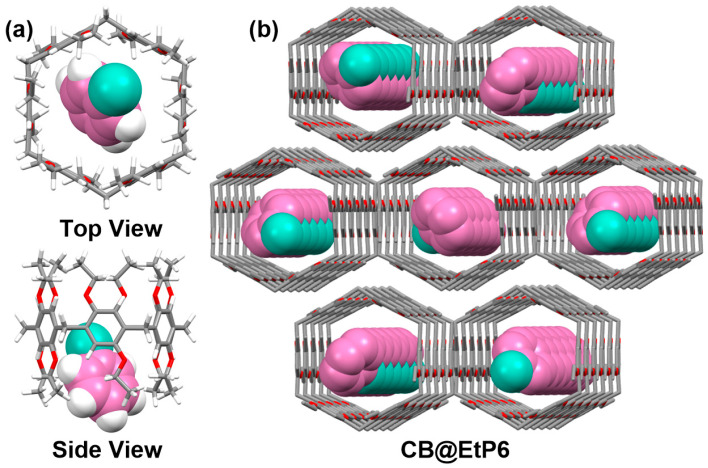
Single crystal of **CB**@**EtP6**: (**a**) top view and side view; (**b**) packing mode along the *a* axis (H atoms were omitted for clarity).

In order to study the adsorption mechanism of **EtP6**α for **CB** and **CCH**, we tried to grow single crystals of **CB**@**EtP6** and **CCH**@**EtP6**. Fortunately, the single crystal of **CB**@**EtP6** was obtained by slowly evaporating the **CB** solution of **EtP6** and characterized by single-crystal X-ray diffraction analysis. One **CB** molecule was located in the cavity of **EtP6** to form a 1:1 host–guest complex, as demonstrated by the single-crystal structure of **CB**@**EtP6** (Figure 4a). The C–H···O interaction between an O atom on the ethoxy group of **EtP6** and an H atom of **CB** stabilized **CB**@**EtP6** (Figure S17) [61,62,63]. Additionally, hexagonal **EtP6** molecules were able to pack into window-to-window patterns, which promoted the formation of honeycomb-like infinite edge-to-edge 1D channels (Figure 4b). However, the single-crystal structure of **CCH**@**EtP6** could not be obtained by various methods, so we establish and optimize the structural model of **CCH**@**EtP6**. Similarly, **CCH** was also located in the center of the cavity of **EtP6**, forming a 1:1 host–guest complex (Figure S18). By measuring the interatomic distances, we estimated that **CCH**@**EtP6** was stabilized by the C–H···Cl interaction between an H atom on the ethoxy group of **EtP6** and a Cl atom of **CCH**, C–H···π interactions between benzene rings of **EtP6** and H atoms of **CCH**, and by C–H···O interactions between O atoms on the ethoxy group of **EtP6** and H atoms of **CCH** (Figure S19). These results suggested that the main driving force for the adsorption process came from non-covalent interactions (C–H···Cl, C–H···π, C–H···O) between **EtP6** and guests.

In addition, the thermodynamics (Gibbs free energies and binding energies) of the formation of host–guest complexes (**CB**@**EtP6** and **CCH**@**EtP6**) were calculated using density functional theory (DFT) [64,65,66,67], and the mechanism of the selective adsorption of **CCH** vapor by **EtP6**α was explained (Table 1 and Table S2). The Gibbs free energies and binding energies were calculated using the following equations:

Δ*G* = *G*_host–guest_ − *G*_host_ − *G*_guest_, *E*_BE_ = *E*_host–guest_ − *E*_host_ − *E*_guest_ (1)

The Gibbs free energies of **CB**@**EtP6** and **CCH**@**EtP6** were calculated to be −56.27 kJ/mol and −82.84 kJ/mol, respectively. Compared with **CB**@**EtP6**, **CCH**@**EtP6** had a lower Gibbs free energy. These results revealed that the adsorption of **EtP6**α for both **CB** vapor and **CCH** vapor was spontaneous, but the adsorption process of **EtP6**α for **CCH** vapor was more prone to occur. In addition, the binding energy of **CCH**@**EtP6** was −142.00 kJ/mol, which was lower than that of **CB**@**EtP6** (−113.17 kJ/mol), indicating that **CCH**@**EtP6** was more stable than **CB**@**EtP6**.

## 3. Materials, Theoretical Calculations, and Methods

### 3.1. Materials

All chemicals, including chlorobenzene (**CB**) and chlorocyclohexane (**CCH**), were purchased and used as received. Perethylated pillar[6]arene (**EtP6**) was synthesized as described previously [32]. Activated crystalline EtP6 (**EtP6***α*) was recrystallized from acetone and dried under vacuum at 120 °C overnight.

### 3.2. Theoretical Calculations

All-electron DFT calculations were carried out by Gaussian G09. For geometry optimization and frequency calculations, the BLYP functional and def2-SVP basis set were used, and the optimal geometry for each compound was determined. The singlet point energy calculations were performed with B3LYP functional and a larger def2-TZVP basis set. The weak interaction was corrected by the DFT-D3 dispersion correction with BJ-damping, which improved the calculation accuracy.

### 3.3. Methods

All experimental methods can be found in the Supplementary Materials.

## 4. Conclusions

In summary, we investigate the ability of nonporous adaptive crystals of perethylated pillar[6]arene (**EtP6**α) to separate **CCH** from the mixture of **CB** and **CCH**. **EtP6**α can separate 99.5% of pure **CCH** vapor from the vapor mixture of **CB** and **CCH** (*v*:*v* = 1:1). DFT calculations show that the ability of **EtP6**α to selectively adsorb **CCH** vapor from the vapor mixture of **CB** and **CCH** (*v*:*v* = 1:1) is due to the higher thermodynamic stability of **CCH**-loaded **EtP6** (**CCH**@**EtP6**) than **CB**-loaded **EtP6** (**CB**@**EtP6**). Additionally, **EtP6**α has good recycling performance due to the reversible transformation between the guest-loaded structure and the guest-free structure. Therefore, **EtP6**α is a promising material for the adsorptive separation of aromatic hydrocarbons and cyclic aliphatic compounds. Future research will focus on how to increase the adsorption amount of **EtP6**α to achieve more efficient separation progress; for example, optimizing the crystal structures, adjusting the adsorption conditions, and combining with other porous adsorbents.

## Figures and Tables

**Table 1:** The optimized structures, Gibbs free energies, and binding energies of **CB**@**EtP6** and **CCH**@**EtP6**.

Species	Structures	Δ*G* (kJ/mol)	*E*_BE_ (kJ/mol)
**CB**@**EtP6**	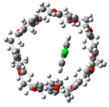	−56.27	−113.17
**CCH**@**EtP6**	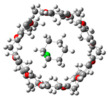	−82.84	−142.00

## Data Availability

Data are contained within the article and Supplementary Materials.

## Supplementary Materials

The following supporting information can be downloaded at: https://www.mdpi.com/article/10.3390/molecules30153312/s1, Figure S1: ^1^H NMR spectrum (600 MHz, CDCl_3_, 293 K) of **EtP6***α*; Figure S2: TGA of **EtP6***α*; Figure S3: PXRD pattern of **EtP6***α*; Figure S4: ^1^H NMR spectrum (600 MHz, CDCl_3_, 293 K) of **EtP6***α* after adsorption of **CB** vapor for 24 h; Figure S5: ^1^H NMR spectrum (600 MHz, CDCl_3_, 293 K) of **EtP6***α* after adsorption of **CCH** vapor for 3 h; Figure S6: ^1^H NMR spectrum (600 MHz, CDCl_3_, 293 K) of (I) **EtP6***α* and (II) **EtP6***α* after adsorption of **CB** vapor; Figure S7: ^1^H NMR spectrum (600 MHz, CDCl_3_, 293 K) of (I) **EtP6***α* and (II) **EtP6***α* after adsorption of **CCH** vapor; Figure S8: TGA of **EtP6***α* after adsorption of **CB** vapor for 24 h; Figure S9: TGA of **EtP6***α* after adsorption of **CCH** vapor for 3 h; Figure S10: PXRD patterns of (I) original **EtP6***α* and (II) **EtP6***α* after adsorption of **CB** vapor; Figure S11: PXRD patterns of (I) original **EtP6***α* and (II) **EtP6***α* after adsorption of **CCH** vapor; Figure S12: ^1^H NMR spectrum (600 MHz, CDCl_3_, 293 K) of **EtP6***α* after adsorption of the vapor mixture of **CB** and **CCH** (*v*:*v* = 1:1) for 3 h; Figure S13: TGA of **EtP6***α* after adsorption of the vapor mixture of **CB** and **CCH** (*v*:*v* = 1:1) for 3 h; Figure S14: PXRD patterns of (I) original **EtP6***α*, (II) **EtP6***α* after adsorption of **CCH** vapor, and (III) **EtP6***α* after adsorption of the vapor mixture of **CB** and **CCH** (*v*:*v* = 1:1); Figure S15: Relative uptakes of **CCH** and **CB** vapors adsorbed by **EtP6***α* for 3 h using HS-GC; Figure S16: ^1^H NMR spectra (600 MHz, CDCl_3_, 293 K) of **EtP6***α* after adsorption of the vapor mixture of **CB** and **CCH** (*v*:*v* = 1:1) for five cycles: (a) original **EtP6***α*; (b) **EtP6***α* after adsorption of the vapor mixture of **CB** and **CCH** (*v*:*v* = 1:1) for the first cycle; (c) **EtP6***α* after desorption of **CCH** for the first cycle; (d) **EtP6***α* after adsorption of the vapor mixture of **CB** and **CCH** (*v*:*v* = 1:1) for the second cycle; (e) **EtP6***α* after desorption of **CCH** for the second cycle; (f) **EtP6***α* after adsorption of the vapor mixture of **CB** and **CCH** (*v*:*v* = 1:1) for the third cycle; (g) **EtP6***α* after desorption of **CCH** for the third cycle; (h) **EtP6***α* after adsorption of the vapor mixture of **CB** and **CCH** (*v*:*v* = 1:1) for the fourth cycle; (i) **EtP6***α* after desorption of **CCH** for the fourth cycle; (j) **EtP6***α* after adsorption of the vapor mixture of **CB** and **CCH** (*v*:*v* = 1:1) for the fifth cycle; Figure S17: Single crystal of **CB**@**EtP6**: illustration of C–H···O interaction between **EtP6** and **CB**; Figure S18: The optimized structure of **CCH**@**EtP6**: (a) top view; (b) side view; Figure S19: The optimized structure of **CCH**@**EtP6**: illustration of C–H···Cl, C–H···π, and C–H···O interactions between **EtP6** and **CCH**; Table S1: Experimental single-crystal X-ray data for **CB**@**EtP6**; Table S2: The optimized structures, Gibbs free energies, and binding energies of **EtP6**, **CB**, **CCH**, **CB**@**EtP6**, and **CCH**@**EtP6**; Table S3: Comparison of the efficiency of separating **CCH** from **CB** for **EtP6***α* with other reported adsorbents. Refs. [68,69,70] are cited in the Supplementary Materials.
