# Supplementary material for: Adsorptive Separation of Chlorobenzene and Chlorocyclohexane by Nonporous Adaptive Crystals of Perethylated Pillar[6]arene
Source: Molecules. 2025 Aug 7;30(15):3312. doi: 10.3390/molecules30153312 (PMC12348486; doi:10.3390/molecules30153312)
Supplement: Supplementary file 1 [file molecules-30-03312-s001.zip › molecules-3705532-supplementary.pdf]

Adsorptive Separation of Chlorobenzene and Chlorocyclohexane by Nonporous Adaptive Crystals of Perethylated Pillar[6]arene

Sha Wu [1,†], Yuyue Chi [1,†], Qian Dong [1] and Jiong Zhou [1,2,*]

[1] Department of Chemistry, College of Sciences, Northeastern University, Shenyang 110819, China; 2200283@stu.neu.edu.cn (S.W.); 2300286@stu.neu.edu.cn (Y.C.); 2270038@stu.neu.edu.cn (Q.D.)
[2] Key Laboratory of Functional Molecular Solids, Ministry of Education, School of Chemistry and Materials Science, Anhui Normal University, Wuhu 241002, China
* Correspondence: zhoujiong@mail.neu.edu.cn
† These authors contributed equally to this work.

Supplementary Information (16 Pages)

1. *Materials* S2
2. *Methods* S2
3. *Crystallography data* S3
4. *Characterization of nonporous adaptive crystals of* ***EtP6*** *(****EtP6****α)* S4
5. *Single-component adsorption experiments* S5
6. *Selectivity analysis of* ***EtP6****α for the vapor mixture of* ***CB*** *and* ***CCH*** S9
7. *Recyclability of* ***EtP6****α* S12
8. *Non-covalent interaction analysis in single crystal structure of* ***CB@EtP6*** S13
9. *Computational analysis* S13
10. *References* S16

1. *Materials*

All chemicals, including chlorobenzene (**CB**) and chlorocyclohexane (**CCH**), were purchased and used as received. Perethylated pillar[6]arene (**EtP6**) was synthesized as described previously.[S1,S2] Activated crystalline **EtP6** (**EtP6**α) was recrystallized from acetone and dried under a vacuum at 120 °C overnight.

2. *Methods*

2.1. Solution nuclear magnetic resonance

Solution nuclear magnetic resonance (^{1}H NMR) spectra were recorded at 600 MHz using a Bruker Avance 600 NMR spectrometer.

2.2. Powder X-ray diffraction

Powder X-ray diffraction (PXRD) data were collected on a Rigaku Ultimate-IV X-ray diffractometer operating at 40 kV/30 mA using the Mo Kα line (λ = 1.5418 Å). Data were measured over the range of 5−40° in 5°/min steps over 7 min.

2.3. Thermogravimetric analysis

Thermogravimetric analysis (TGA) was carried out using a Q5000IR analyzer (TA Instruments) with an automated vertical overhead thermobalance. The samples were heated at 10 °C/min using N_2 as the protective gas.

2.4. Single crystal growth

Single crystals of **CB@EtP6** was grown by slow evaporation: 3.00 mg of dry **EtP6**α powders were put in a small vial where 1 mL of **CB** solution was added. Then the mixture was oscillated constantly until all **EtP6**α powders were dissolved. The resultant transparent solution was allowed to evaporate slowly to give nice colorless crystals in 2 to 3 days.

2.5. Single crystal X-ray diffraction

Single crystal X-ray diffraction data were collected on a Bruker D8 VENTURE CMOS X-ray diffractometer with graphite monochromated Mo Kα radiation (λ = 0.71073 Å).

2.6. Head space gas chromatography

Head space gas chromatography (HS-GC) Analysis: HS-GC measurements were carried out using an Agilent 7890B instrument configured with an FID detector and a DB-624 column (30 m × 0.53 mm × 3.0 μm). Samples were analyzed using headspace injections and were performed by incubating the sample at 100 °C for 10 min followed by sampling 1 mL of the headspace. The following HS-GC method was used: the oven was programmed from 50 °C, and ramped in 10 °C min^{-1} increments to 150 °C with a 15 min hold; the total run time was 25 min; the injection temperature was 250 °C; the detector temperature was 280 °C with nitrogen, air, and make-up flow rates of 35, 350 and 35 mL min^{-1}, respectively; the helium (carrier gas) flow rate was 3.0 mL min^{-1}. The samples were injected in split mode (30:1).

3. Crystallography data

Table S1. Experimental single crystal X-ray data for **CB@EtP6**.

Formula	**CB@EtP6**
Crystallization Solvent	chlorobenzene
Collection Temperature (K)	300.00
Formula	$C_{72}H_{89}ClO_{12}$
Formula Weight	1181.88
Crystal System	Orthorhombic
Space Group	Fddd
a [Å]	12.5116(12)
b [Å]	26.760(3)
c [Å]	45.864(4)
α [º]	90
β [º]	90
γ [º]	90
V [Å^3]	15355(3)
Z	8
D_{calcd} [g cm^{-3}]	1.022
Absorption coefficient (mm^{-1})	0.855
F(000)	5072
Crystal size/mm^3	0.120 × 0.100 × 0.080
Radiation	MoKα (λ = 0.71073)
Theta range/°	4.018 to 66.499
Index ranges	-12 <= h <= 14, -30 <= k <= 31, -48 <= l <= 54
Reflections collected	29226
Independent reflections	3394 [R_{int} = 0.1612]
Data/restraints/parameters	3394/105/231
CCDC	2408714

*4. Characterization of nonporous adaptive crystals of **EtP6** (**EtP6**α)*

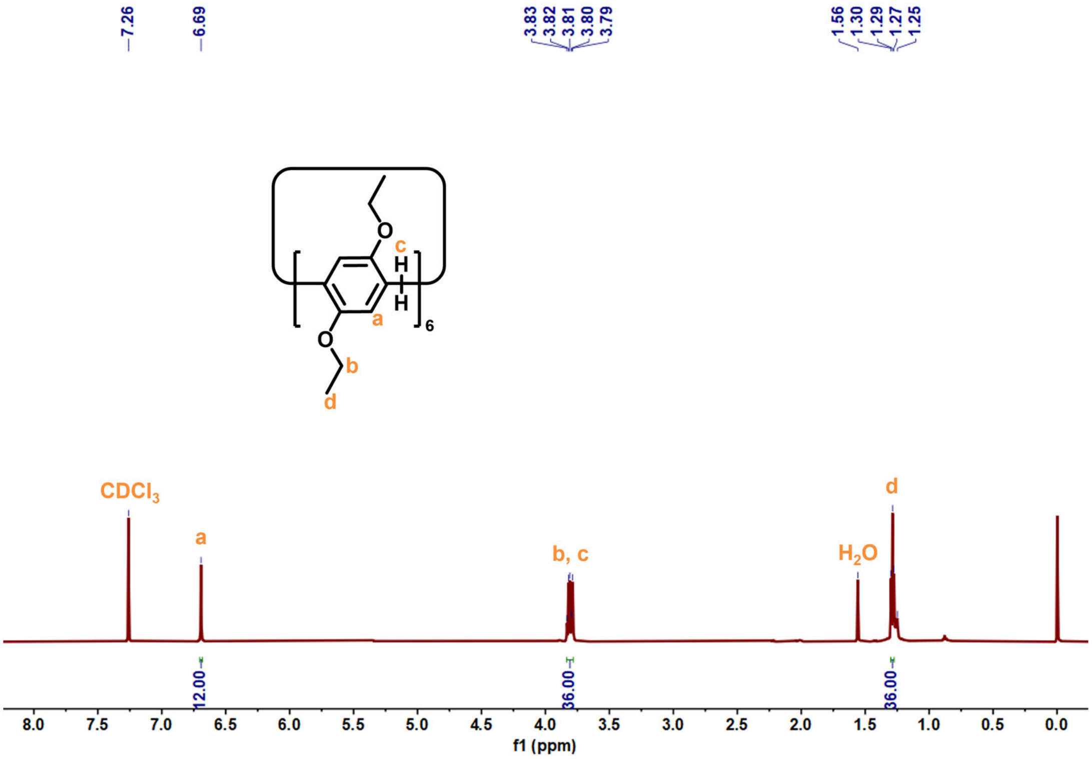

Figure S1. ^{1}H NMR spectrum (600 MHz, $CDCl_3$, 293 K) of **EtP6**α.

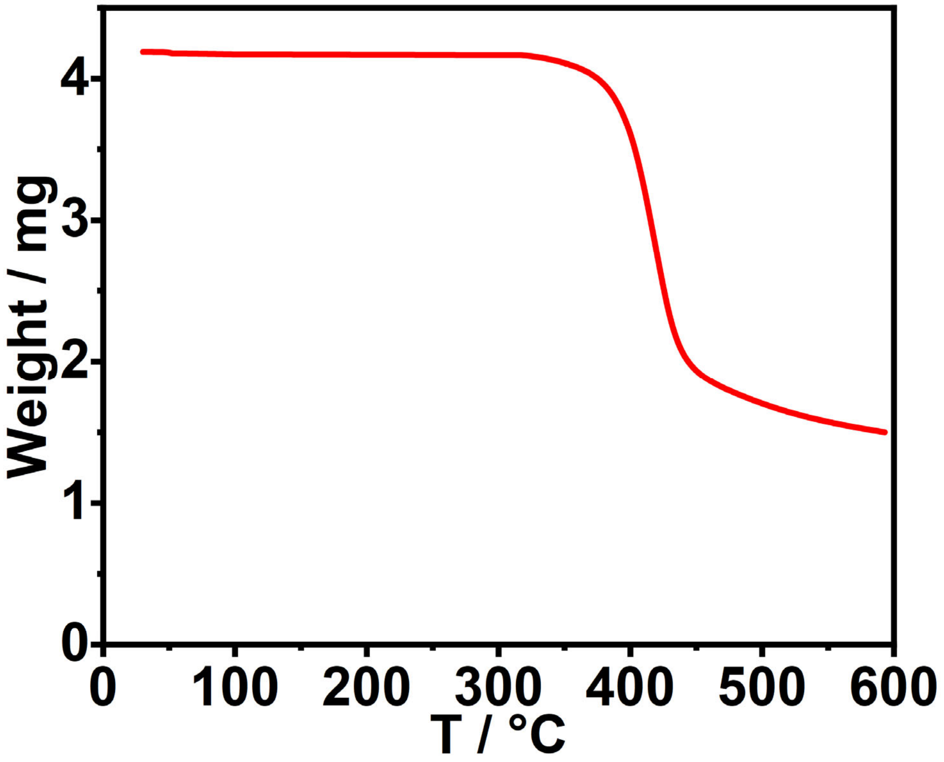

Figure S2. TGA of **EtP6**α.

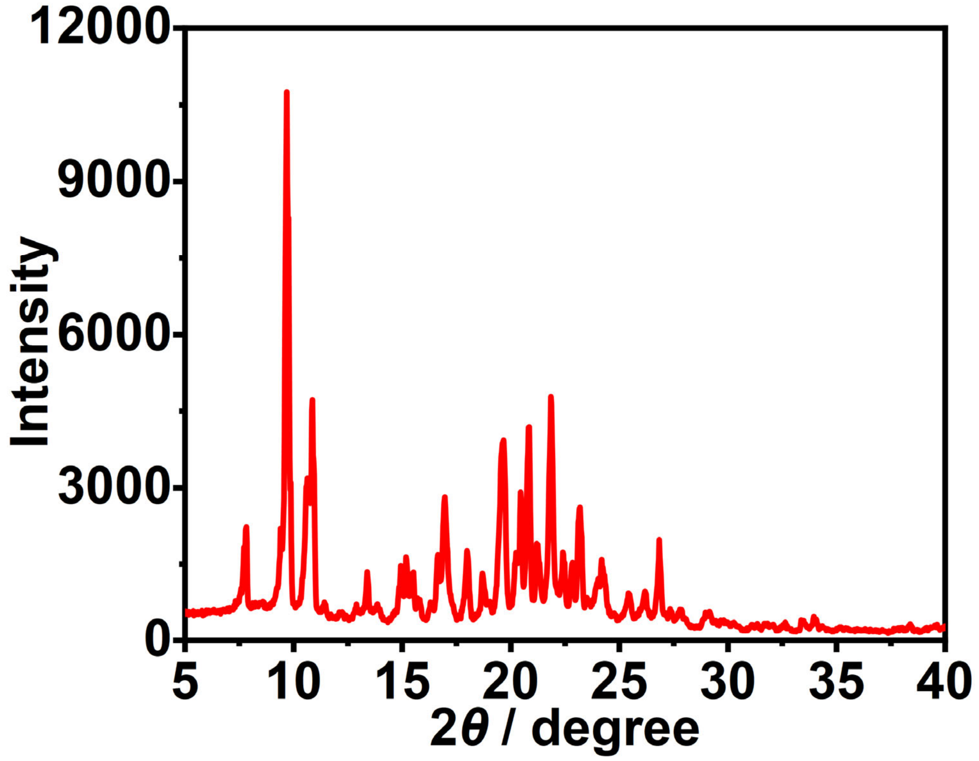

Figure S3. PXRD pattern of **EtP6**α.

5. Single-component adsorption experiments

^{1}H NMR experiments were performed by dissolving **EtP6**α after the adsorption of single-component **CB** vapor and **CCH** vapor in $CDCl_3$. TGA profiles were recorded using **EtP6**α after the adsorption of single-component **CB** vapor and **CCH** vapor.

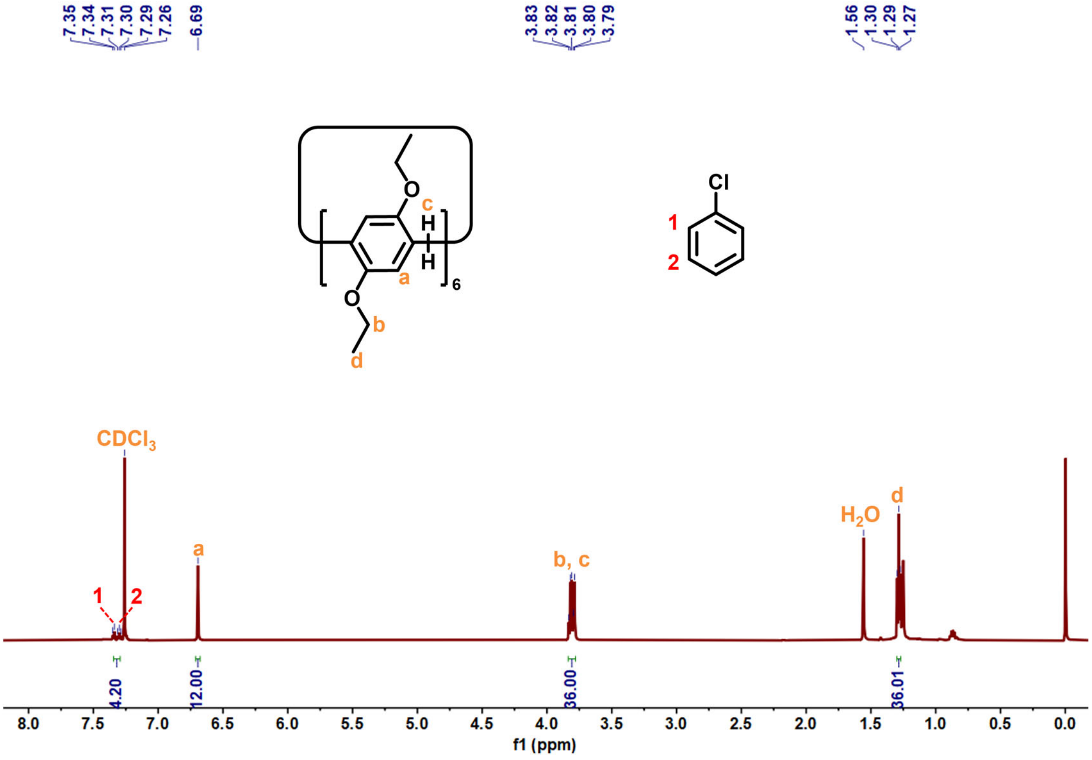

Figure S4. ^{1}H NMR spectrum (600 MHz, $CDCl_3$, 293 K) of **EtP6**α after adsorption of **CB** vapor for 24 h.

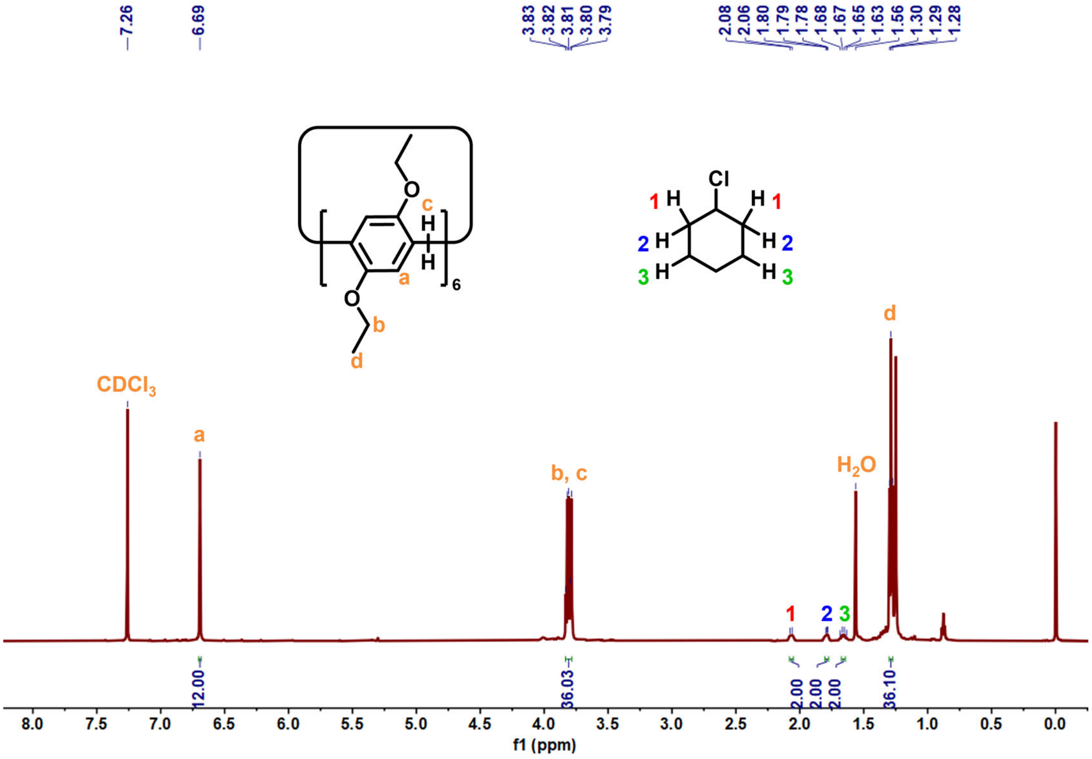

Figure S5. ^{1}H NMR spectrum (600 MHz, $CDCl_3$, 293 K) of **EtP6**α after adsorption of **CCH** vapor for 3 h.

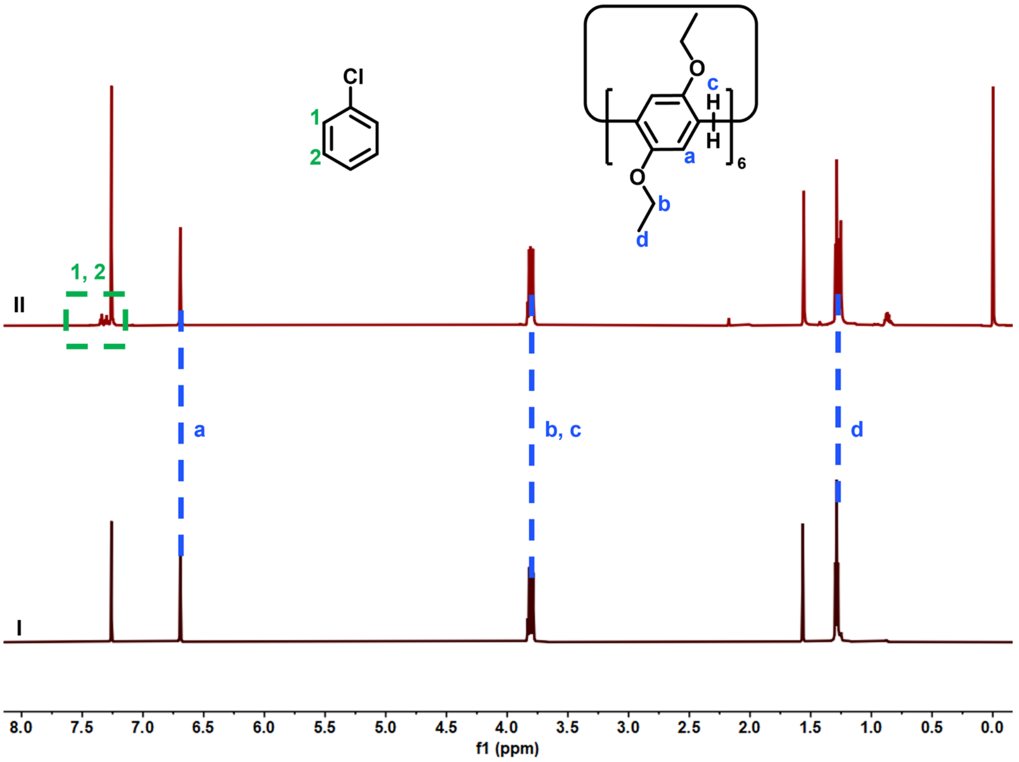

Figure S6. ^{1}H NMR spectrum (600 MHz, $CDCl_3$, 293 K) of (I) **EtP6**α and (II) **EtP6**α after adsorption of **CB** vapor.

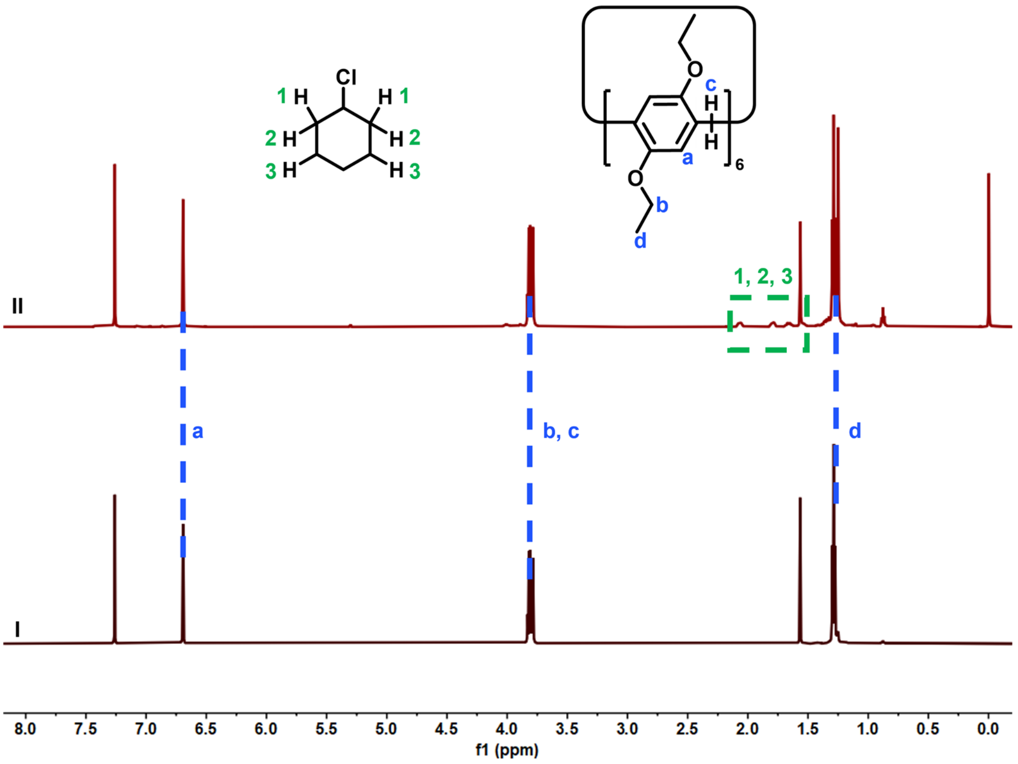

Figure S7. ^{1}H NMR spectrum (600 MHz, $CDCl_3$, 293 K) of (I) **EtP6*α*** and (II) **EtP6*α*** after adsorption of **CCH** vapor.

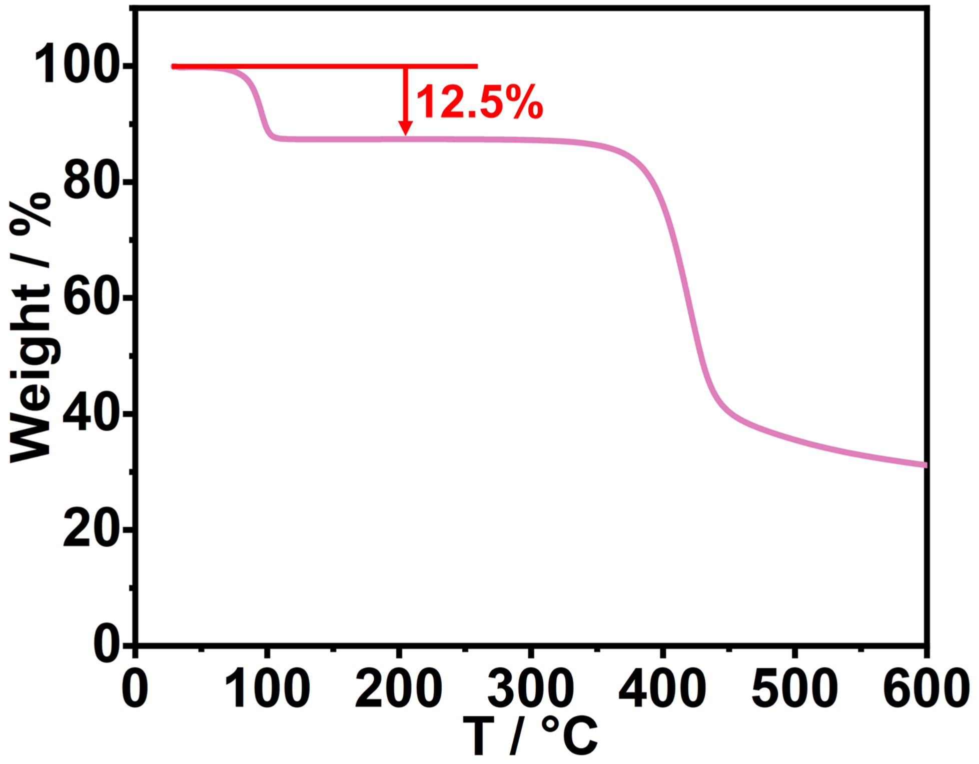

Figure S8. TGA of **EtP6*α*** after the adsorption of **CB** vapor for 24 h. The weight loss at 90 °C can be calculated as about one **CB** molecule per **EtP6** molecule.

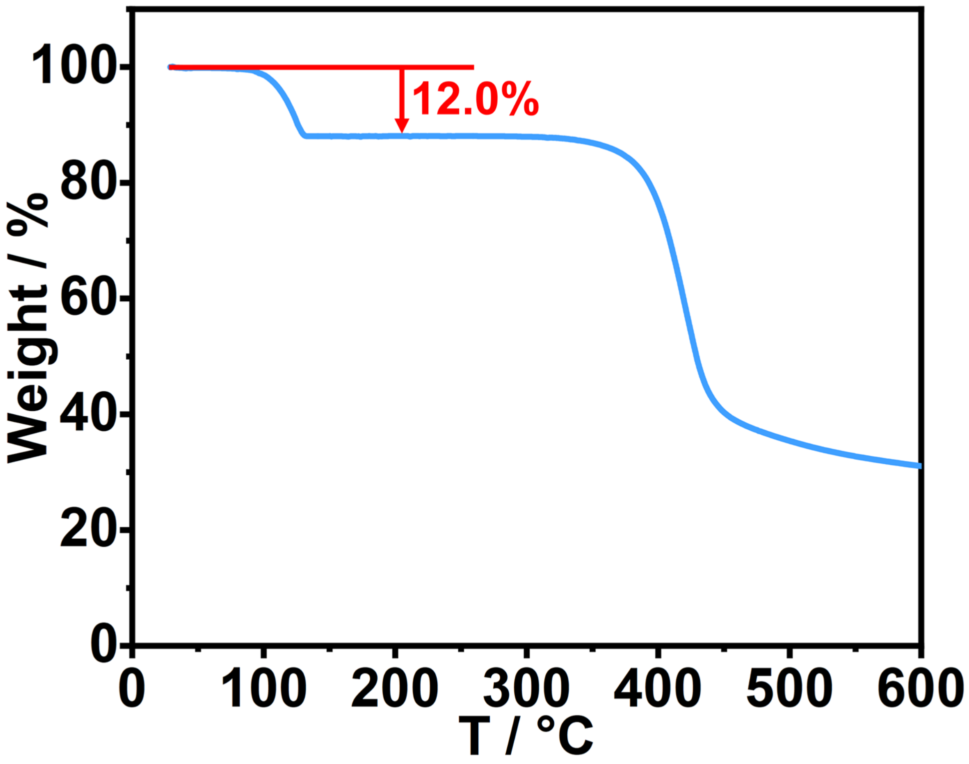

Figure S9. TGA of **EtP6**α after the adsorption of **CCH** vapor for 3 h. The weight loss at 90 °C can be calculated as about one **CCH** molecule per **EtP6** molecule.

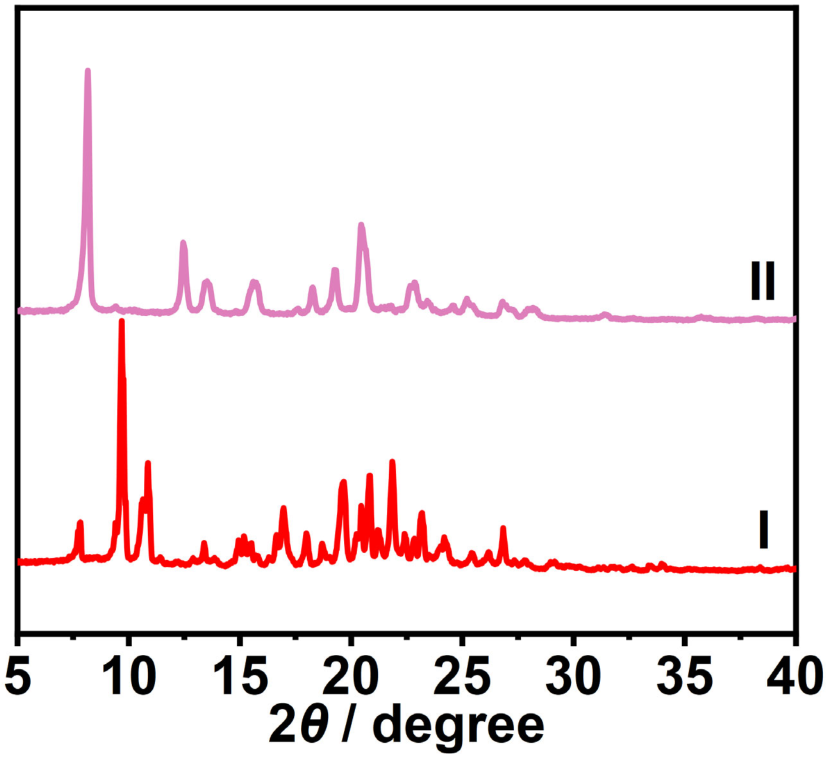

Figure S10. PXRD patterns of (I) original **EtP6**α, and (II) **EtP6**α after adsorption of **CB** vapor.

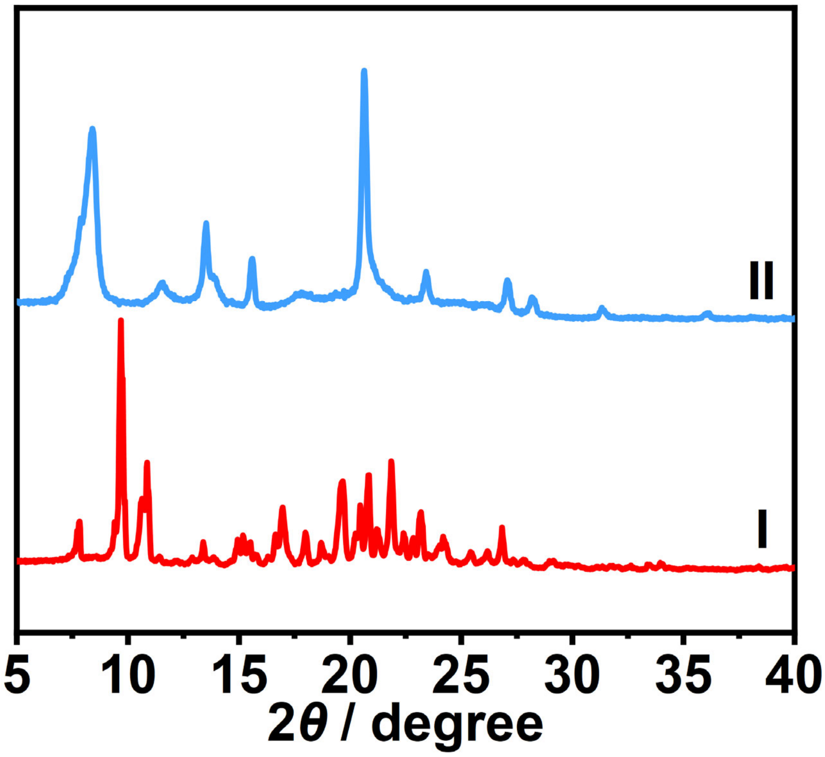

Figure S11. PXRD patterns of (I) original **EtP6*α***, and (II) **EtP6*α*** after adsorption of **CCH** vapor.

*6. Selectivity analysis of **EtP6α** for the vapor mixture of **CB** and **CCH***

An open 5 mL vial containing 2.00 mg of guest-free **EtP6*α*** was placed in a sealed 20 mL vial containing 1 mL of the liquid mixture of **CB** and **CCH** (v:v = 1:1). Uptake in **EtP6*α*** was measured hour by hour by completely dissolving the crystals and measuring the ratio of **CB** or **CCH** to **EtP6*α*** by ^{1}H NMR. The relative uptakes of **CB** vapor and **CCH** vapor in **EtP6*α*** were also measured by heating the crystals to release the adsorbed vapor and detecting the relative amounts of **CB** and **CCH** in the released vapor using HS-GC.

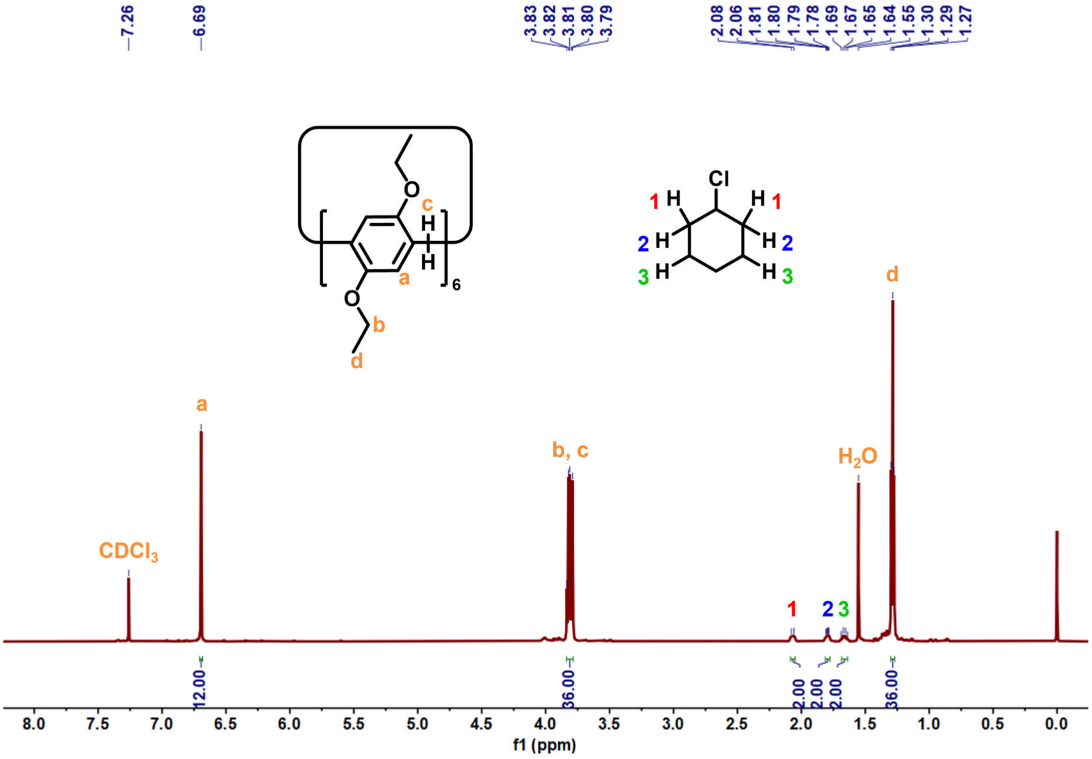

Figure 12. ^{1}H NMR spectrum (600 MHz, $CDCl_3$, 293 K) of **EtP6***α* after the adsorption of the vapor mixture of **CB** and **CCH** (*v*:*v* = 1:1) for 3 h.

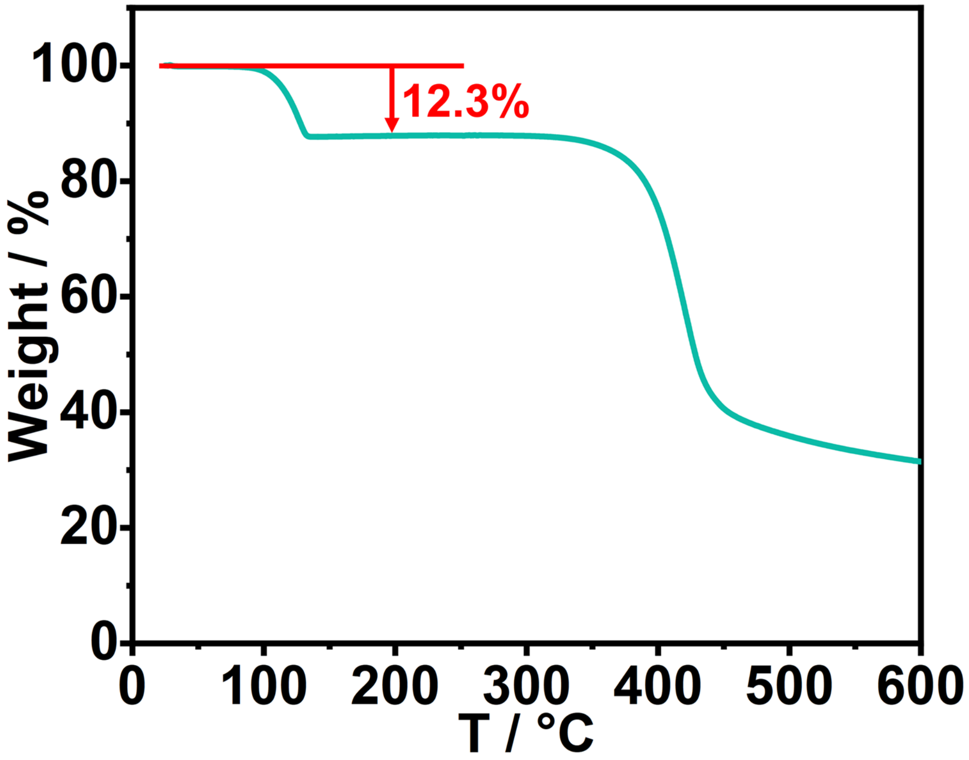

Figure 13. TGA of **EtP6***α* after the adsorption of the vapor mixture of **CB** and **CCH** (*v*:*v* = 1:1) for 3 h. The weight loss at 90 °C can be calculated as about one **CCH** molecule per **EtP6** molecule.

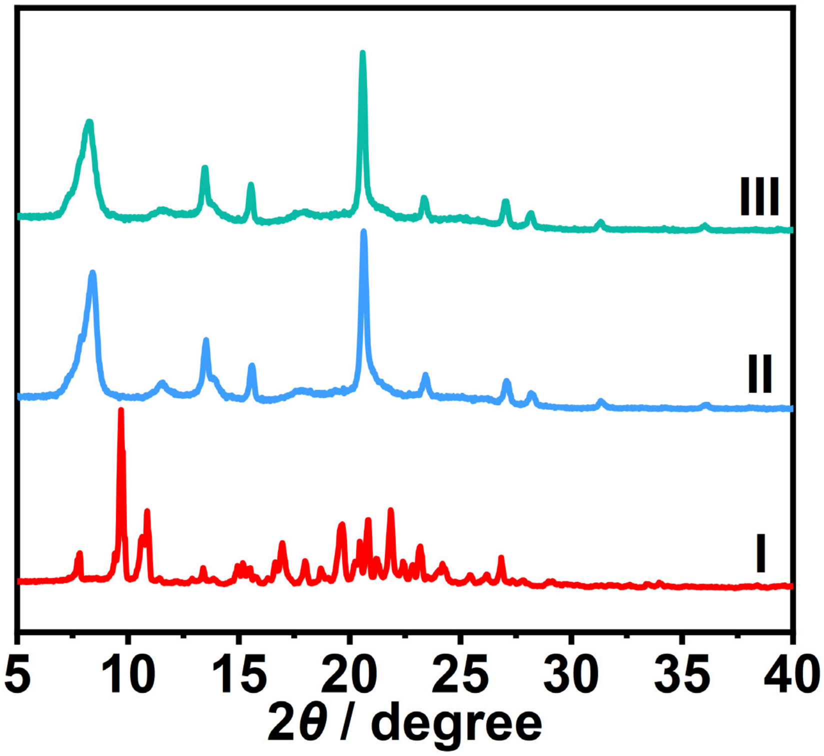

Figure 14. PXRD patterns of (I) original **EtP6*α***, (II) **EtP6*α*** after the adsorption of **CCH** vapor, and (III) **EtP6*α*** after adsorption of the vapor mixture of **CB** and **CCH** (*v*:*v* = 1:1).

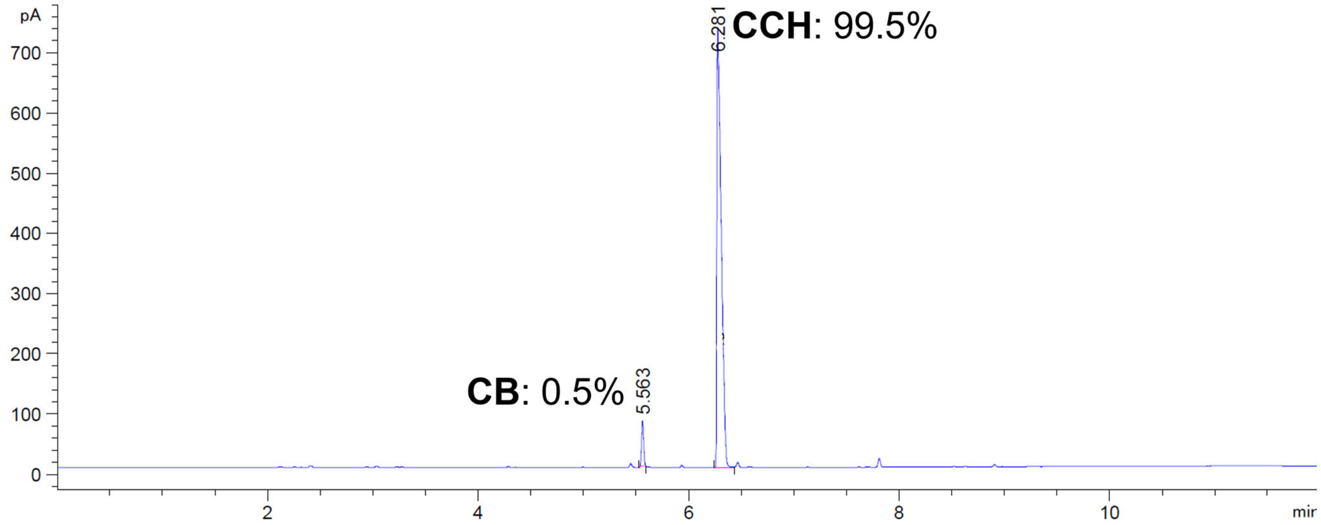

Figure S15. Relative uptakes of **CCH** and **CB** vapors adsorbed by **EtP6*α*** for 3 h using HS-GC.

*7. Recyclability of **EtP6α***

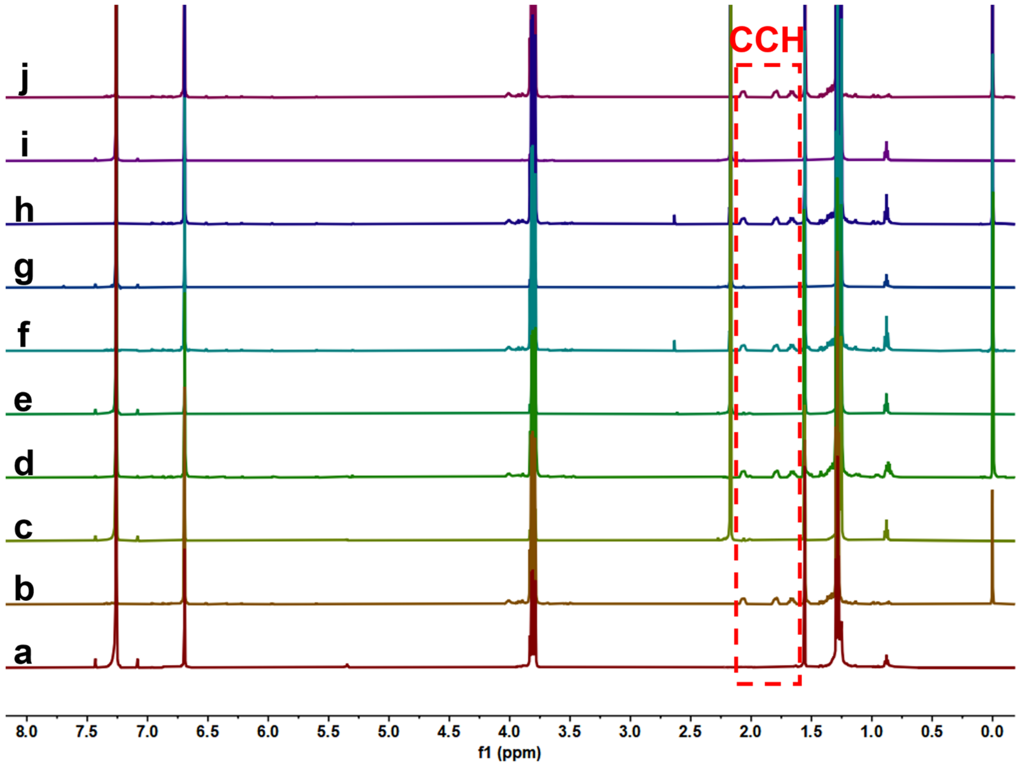

Figure S16. ^{1}H NMR spectra (600 MHz, $CDCl_3$, 293 K) of **EtP6*α*** after the adsorption of the vapor mixture of **CB** and **CCH** (*v*:*v* = 1:1) for five cycles: (a) Original **EtP6*α***; (b) **EtP6*α*** after the adsorption of the vapor mixture of **CB** and **CCH** (*v*:*v* = 1:1) for the first cycle; (c) **EtP6*α*** after the desorption of **CCH** for the first cycle; (d) **EtP6*α*** after adsorption of the vapor mixture of **CB** and **CCH** (*v*:*v* = 1:1) for the second cycle; (e) **EtP6*α*** after the desorption of **CCH** for the second cycle; (f) **EtP6*α*** after the adsorption of the vapor mixture of **CB** and **CCH** (*v*:*v* = 1:1) for the third cycle; (g) **EtP6*α*** after the desorption of **CCH** for the third cycle; (h) **EtP6*α*** after the adsorption of the vapor mixture of **CB** and **CCH** (*v*:*v* = 1:1) for the fourth cycle; (i) **EtP6*α*** after the desorption of **CCH** for the fourth cycle; (j) **EtP6*β*** after the adsorption of the vapor mixture of **CB** and **CCH** (*v*:*v* = 1:1) for the fifth cycle.

*8. Non-covalent interactions analysis in single crystal structure of **CB@EtP6***

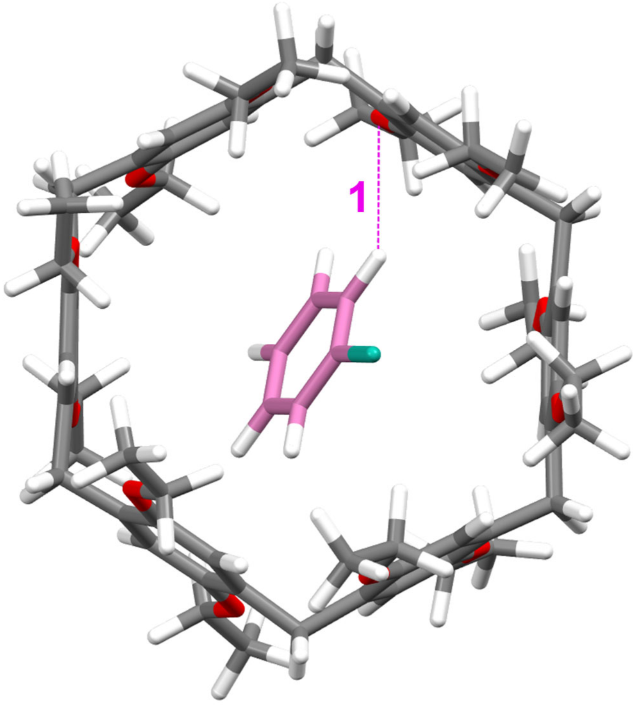

Figure S17. Single crystal of **CB@EtP6**: illustration of C–H···O interaction between **EtP6** and **CB**.

9. Computational analysis

All-electron DFT calculations were carried out by Gaussian G09. [S3,S4]

For geometry optimization and frequency calculations, the BLYP functional and def2-SVP basis set were used, and the optimal geometry for each compound was determined. The singlet point energy calculations were performed with B3LYP functional and a larger basis set def2-TZVP basis set.

The weak interaction was corrected by the DFT-D3 dispersion correction with BJ-damping, which improved the calculation accuracy.

Then, the binding energy (E_{BE}) was calculated by the following formula:

$$E_{\mathrm{BE}} = E_{\mathrm{host+guest}} - E_{\mathrm{host}} - E_{\mathrm{guest}}$$

where E_{host} was the singlet point energy of **EtP6**, E_{guest} was the singlet point energy of **CB** or **CCH**, and $E_{\mathrm{host+guest}}$ was the singlet point energy of the host−guest complex.

The single point energy of each compound was added to the free energy correction terms calculated before to obtain the Gibbs free energy.

Then, the Gibbs free energy change (ΔG) of the binding process was calculated by the following formula:

$$\Delta G = G_{\mathrm{host+guest}} - G_{\mathrm{host}} - G_{\mathrm{guest}}$$

where G_{host} represented the Gibbs free energy of **EtP6**, G_{guest} represented the Gibbs free energy of **CB** or **CCH**, and $G_{host+guest}$ represented the Gibbs free energy of the host−guest complex.

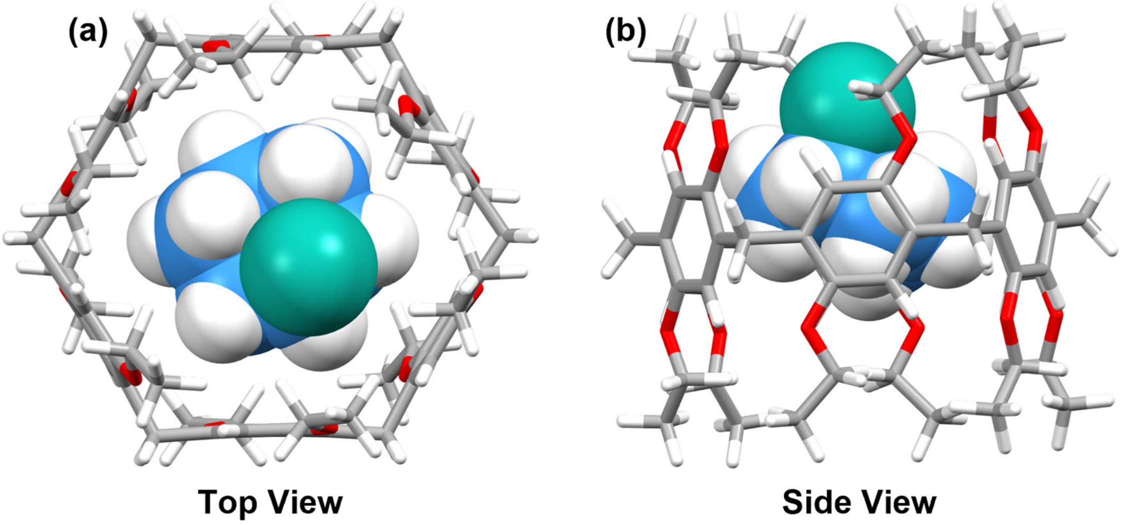

Figure S18. The optimized structure of **CCH@EtP6**: (a) top view; (b) side view.

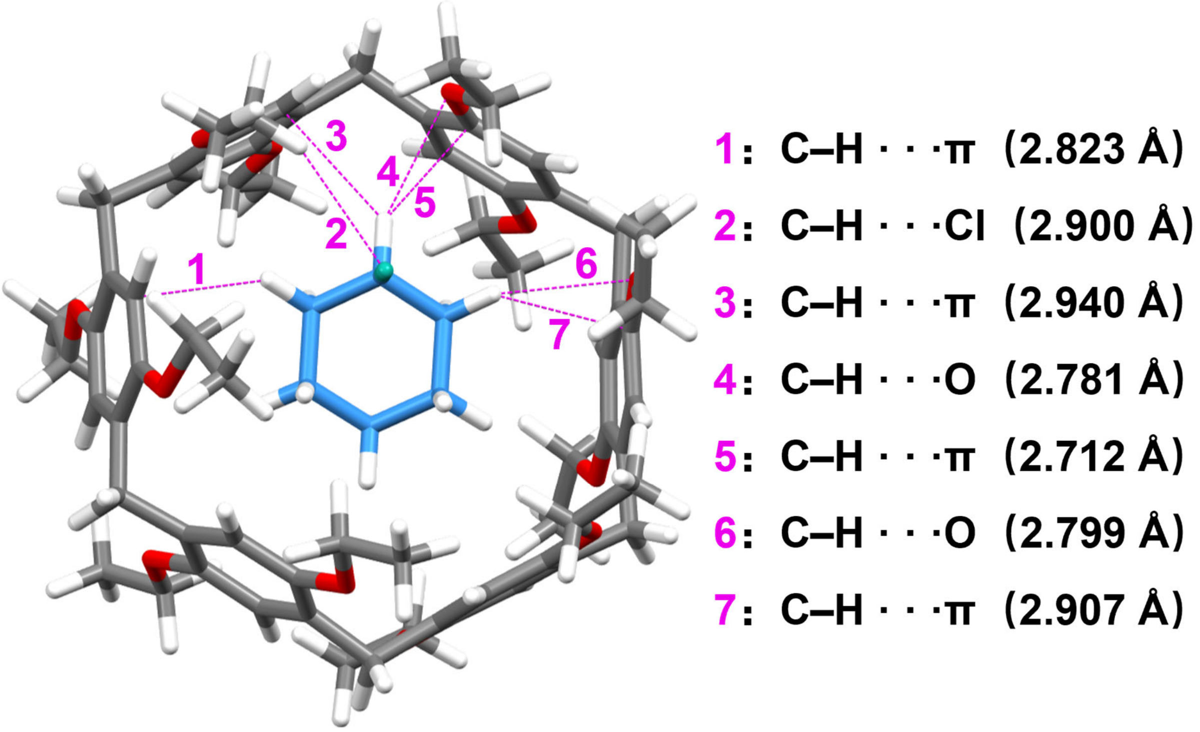

Figure S19. The optimized structure of **CCH@EtP6**: illustration of C–H···Cl, C–H···π and C–H···O interactions between **EtP6** and **CCH**.

Table S2. The optimized structures, Gibbs free energies and binding energies of **EtP6**, **CB**, **CCH**, **CB@EtP6** and **CCH@EtP6**.

Species	Structures	G (kcal/mol)	ΔG (kJ/mol)	E (kcal/mol)	E_{BE} (kJ/mol)
**EtP6**	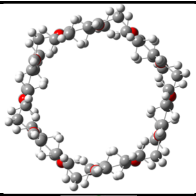	-2175812.28	\	-2176609.90	\
**CB**	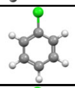	-434114.93	\	-434153.53	\
**CCH**	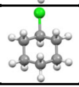	-436357.28	\	-436439.74	\
**CB@EtP6**	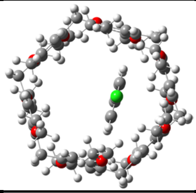	-2609940.64	-56.27	-2610790.40	-113.17
**CCH@EtP6**	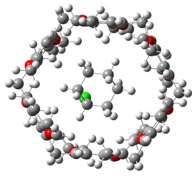	-2612189.33	-82.84	-2613083.53	-142.00

Table S3. Comparison of the efficiency of separating **CCH** from **CB** for **EtP6***α* with other reported adsorbents.

Species	Structures	The efficiency of separating **CCH** from **CB**	Ref.
**Geminiarene (GA*α*)**	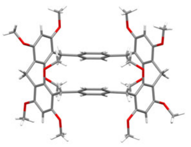	97.5%	S5
**Pillar[6]arene (EtP6*α*)**	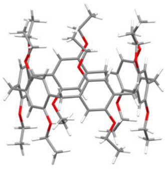	99.5%	This work

10. References

S1. Wang, M.; Zhou, J.; Li, E.; Zhou, Y.; Li, Q.; Huang, F. Separation of monochlorotoluene isomers by nonporous adaptive crystals of perethylated pillar[5]arene and pillar[6]arene. *J. Am. Chem. Soc.* **2019**, *141*, 17102–17106.

S2. Wang, M.; Fang, S.; Yang, S.; Li, Q.; Khashab, N.M.; Zhou, J.; Huang, F. Separation of ethyltoluene isomers by nonporous adaptive crystals of perethylated and perbromoethylated pillararenes. *Mater. Today Chem.* **2022**, *24*, 100919.

S3. Lu, T.; Chen, F. Multiwfn: A multifunctional wavefunction analyzer. *J. Comput. Chem.* **2012**, *33*, 580–592.

S4. Lu, T. A comprehensive electron wavefunction analysis toolbox for chemists, Multiwfn. *J. Chem. Phys.* **2024**, *161*, 082503.

S5. Wu, J.-R.; Yang, Y.-W. Geminiarene: Molecular scale dual selectivity for chlorobenzene and chlorocyclohexane fractionation. *J. Am. Chem. Soc.* **2019**, *141*, 12280–12287.
